# “Give My Daughter the Shot!”: A Content Analysis of the Depiction of Patients with Cancer Pain and Their Management in Hollywood Films

**DOI:** 10.3390/curroncol29110648

**Published:** 2022-10-29

**Authors:** Karim Mukhida, Sina Sedighi, Catherine Hart

**Affiliations:** 1 Department of Anesthesiology, Pain Management and Perioperative Medicine, Faculty of Medicine, Dalhousie University, Halifax, NS B3H 4R2, Canada; 2 Faculty of Medicine, Dalhousie University, Halifax, NS B3H 4R2, Canada; 3 Novagevity, Halifax, NS B3H 0A8, Canada

**Keywords:** cancer, pain, film, cinemeducation, Hollywood

## Abstract

Introduction: Cinemeducation, the pedagogical use of films, has been used in a variety of clinical disciplines. To date, no studies have looked at the use of film depictions of cancer pain and its management in clinical education. We investigated how patients with cancer pain and their management are depicted in Hollywood films to determine whether there is content that would be amenable to use for cancer pain assessment and management education. Methods: A qualitative content analysis was performed. Films that contained characters with or references to cancer pain were searched for using the International Movie Database, the Literature Arts Medicine Database, the History of Medicine and Medical Humanities Database, and Medicine on Screen. After review, 4 films were identified for review and analysis. Results: Themes that emerged from the analysis concerned the films’ depictions of characters with pain, their healthcare providers, the therapies used for pain management, and the setting in which pain management was provided. Conclusions: This study demonstrates that patients with cancer pain are depicted in a compassionate manner. Pain management focused on the use of opioids. The settings in which patients received pain management was depicted as not being amenable to providing holistic care. This variety of topics related to pain management covered in the films make them amenable to use in cinemeducation. This study therefore forms the basis for future work developing film-based cancer education modules.

## 1. Introduction

Pain is frequently associated with cancer and is known to have significant effects on patients’ quality of life [1]. Deficiencies in healthcare professional trainees’ knowledge related to the assessment and management of cancer-related pain have been recognized and are thought to be barriers to improving the quality of patients’ pain management [2]. Studies have found that healthcare professionals may lack sufficient awareness of the problem of cancer pain [3,4], have demonstrated knowledge deficits regarding cancer pain management [1,4,5,6,7], and have inappropriate concerns about issues such as addiction, tolerance, and dependence [3,4,8]. Lack of adequate cancer pain education is thought to contribute to less-than-optimal pain management [6]. Healthcare professional trainees have been shown to have insight into these educational deficiencies; they have therefore desired additional training in pain management for and communication with patients with cancer or terminal illnesses as well as addressing other contextual and psychosocial issues related to pain [6,9]. 

The International Association for the Study of Pain (IASP) recognized deficiencies in pain management education and therefore developed a subcommittee on Medical School Courses and Curriculum [10]. Nevertheless, pain education in health science curricula remains “disorganized, unstructured, [and] fragmented” [11] (p. 39) with many medical schools not covering any of the IASP core topics [12]. For example, a cross-sectional survey of undergraduate health programs in major universities found that pain education comprised less than 1% of program hours for some disciplines [13]. In Canada, Watt-Watson and colleagues surveyed the entry-to-practice competency requirements that related to pain knowledge, skill, and/or judgement for health professions [14]. No specific competencies were required for medical graduates, which contrasted with veterinary colleges that specified competencies related to pain assessment and management and clearly described the criteria for evaluating pain-related knowledge, skills, and judgement [14]. Additionally, a survey of pain curricula in Canadian health science faculties found that veterinary schools allocated significantly more time to pain education over the course of their programs compared to medical schools (mean 87 h versus 16 h, respectively) [15]. It is therefore not surprising then that studies have found that most medical students and residents surveyed found that their pain education was “poor” or “fair” at best [16] (p. 102) [17] and considered themselves ill-prepared to deal with patients’ suffering [18]. 

Improving health professionals’ pain education has been identified as an important step to improve their comfort and skill in managing pain [19,20]. Medical students’ and residents’ lack of knowledge of pain assessment and management contributes to negative attitudes towards patients with pain [16,21,22,23]. The introduction of pain education in medical school and residency has been shown to improve trainees’ knowledge of pain assessment and treatment, dispel their misconceptions about patients with pain, and improve their attitudes and self-reported skills [24,25,26]. In terms of pain curriculum content, many programs have focused on the biomedical aspects of pain, such as therapeutics [13]. However, it has been recommended that pain curricula need to better address the psychosocial aspects of pain [27,28,29]. Surveys of pain management experts, including the leadership of the American Academy of Pain Medicine, have highlighted the importance of incorporating training in communication and empathy in pain curricula [28,29]. Carr and Bradshaw [27] liken the standard pain curriculum to “an approach to driver education in which classes in combustion chemistry and automechanics were mandatory, while on-road training in courtesy and defensive driving amidst traffic were optional” (p. 14). They suggest “flipping the curriculum” so that it takes a sociopsychobiological approach to education that will better prepare trainees to manage the complex economic, psychological, and social effects of pain on patients’ lives and not merely regard them as epiphenomena [27]. Furthermore, pain education curricula need to better address the affective component of the pain experience [30] and trainees communication skills [20]. More innovative ways of delivering pain education have been requested by educators and trainees alike [15,31]. Instead of the typical didactic, lecture-based format that has comprised the majority of curricula, it has been envisioned that programs that incorporate workshops, multidisciplinary panels, small group discussions, and case studies that integrate theory with clinical practice would be more effective in conveying a sociopsychobiological approach to pain education [12,32]. 

Incorporation of the humanities in pain curricula is a unique manner of addressing those curricular deficiencies. Narratives have been used as forms of case studies in order to make trainees more aware of patients’ perspectives, to better understand what patients’ medical problems mean to them, and to help promote a patient-centred approach to clinical care [33,34,35]. Kumagai [34] argues that narratives are particularly well-suited to education curricula because they “offer a glimpse into the subjective experience of illness; they offer entry into the ‘kingdom of the sick’…and, in doing so, provide a complementary perspective to the biomedical knowledge acquired through the study of disease processes” (p. 655). 

The visual arts can be used to improve trainees’ clinical training by honing observational skills and providing a means for trainees to reflect on both the experiences of others and themselves [36]. Films have been used as an educational tool in a variety of clinical disciplines [37,38,39,40,41,42,43,44,45,46,47,48,49,50], such as Family Medicine, Psychiatry, Palliative Care, and Medical Ethics. The term “cinemeducation” has been used to describe “the use of movies on video…for educating…in the psychosocial aspects of medicine” as a tool to facilitate trainees’ better understanding of the way that illness is experienced by patients [37] (p. 92). 

Although the depiction of a variety of medical professionals, conditions, and procedures in films has been studied, to date there have been no studies focusing on the portrayal of characters with cancer pain or their management in films. Lederer has written about cultural representations of cancer in Hollywood films but that analysis did not include discussions of cancer pain *per se* and did not look at contemporary films since it focused on films released between 1930 and 1970 [51]. Pavisic and colleagues focused on psychosocial issues related to pediatric cancer [52]. 

The aim of this study was therefore to explore portrayals of cancer pain and its management in contemporary Hollywood films. Since it has been suggested that films may be used for education purposes, another aim was to determine whether cancer pain-related content could be amenable for use in cinemeducation approaches for medical students and residents to complement other pedagogical strategies. 

## 2. Materials and Methods

### 2.1. Review Design

A qualitative content analysis was performed. First, a list of films was compiled that featured characters with cancer pain or its management independently by two reviewers who searched through the International Movie Database (IMDb) (https://www.imdb.com, 16 July 2020), the Literature Arts Medicine Database (https://medhum.med.nyu.edu/about, 16 July 2020), the History of Medicine and Medical Humanities Databases (https://medhumanities.mcmaster.ca/index/mcmaster-library-collections/resources-themes-bibliogrpahies/history-of-medicine-and-medical-humanities-database, 16 July 2020), and Medicine on Screen (https://medicineonscreen.nlm.nih.gov/category/all/, 16 July 2020). The IMDb is considered a validated tool to search for films for research purposes [52,53,54,55,56]. The following keyword search terms were used: “pain” and “cancer”. These terms were purposefully kept broad to increase the probability that any relevant films were found. The databases were queried for films between January 1989 and January 2020. Rather than reviewing an “exhaustive” list of films that make any reference to cancer pain, the aim followed that described by Flores to consider representative films [57] (p. 657).

### 2.2. Screening of Films

Inclusion criteria included films that depicted characters with cancer pain. Cues from the films were used to determine whether the characters’ pain was cancer-related. Only films releases after 1980 were retained because we were interested in films that might contribute to contemporary attitudes about cancer pain. We included films that were produced by Hollywood film companies or whose directors or actors had a Hollywood connection because we were interested in films that would be more likely to influence public perceptions in North America. Films had to have had a theatrical release or be obtainable on YouTube, Netflix, AmazonPrime, iTunes, or Amazon because included films needed to be those that could easily be accessed by the public. Excluded were children’s movies and animated films because recent studies have already looked at aspects of their depiction of pain [52,58,59]. Documentaries and any television or film series were excluded, as were internet and direct-to-video films. Films that were not in English were also excluded. 

### 2.3. Analysis of Films

Films that met the inclusion criteria were viewed independently in their entireties by two reviewers (KM and SS). Dialogues and scenes related to cancer pain or its management were transcribed or described, respectively, by each reviewer. A qualitative content analysis of the films was performed using approaches described by others for their film analyses [57,58,60,61,62]. Previous studies have created coding schemes for content analysis. After watching the films, reviewers then met to review themes to develop consensus with respect to ideas [57,58,60,61,62]. For our study, reviewers met to compare their transcripts and notes for each film. A coding template was created after initial discussion using an iterative process that included topics related to how characters and their treatment were portrayed. Ideas for themes that emerged with respect to how characters with cancer pain and their pain management were portrayed were first independently noted and then reviewed together to develop consensus with respect to themes, as has been done in other studies [57,58,60,61,62].

## 3. Results

**Figure 1:**
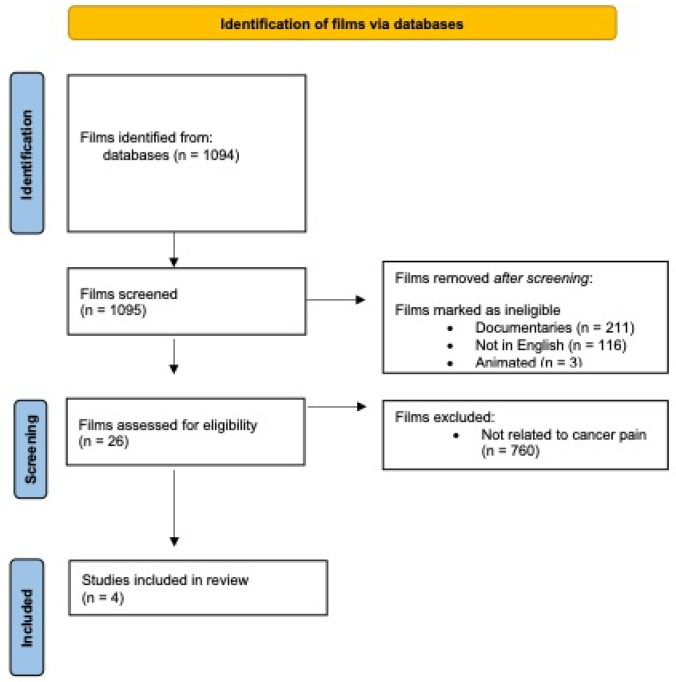
Film management search process.

The search strategy yielded 1094 motion pictures related to cancer. Films were excluded after screening if it was found that they did not deal with cancer-related pain (n = 765), were documentaries (n = 211), were not in English (n = 116), or were found to be animated (n =3). After review, 4 films were identified for review and analysis (Figure 1, Table 1). All were dramas that were made for cinema except for *Wit*, which was made for television. 

The themes that emerged from the qualitative content analysis were categorized into those related to the depiction of: A. characters with cancer pain: the quality of life of characters’ and those close to them as adversely affected by cancer pain; B. healthcare professionals looking after characters with cancer pain: healthcare professionals were out of tune with the needs of characters with pain; C. therapies for pain management: opioids were portrayed as the mainstay of therapy; and D. the settings in which pain management occurred: the environments in which characters with pain are treated did not support holistic management of their pain. 

### 3.1. Depictions of Characters’ Lives with Pain

#### 3.1.1. Cancer Pain as Adversely Affecting Characters’ Quality of Life

A sense of empathy for the life-altering experience that cancer pain entails is elicited in the films. In *Wit*, for example, Vivian Bearing’s life as an English professor ends after she is diagnosed with “advanced metastatic ovarian cancer” (0:50), undergoes chemotherapy “full force” (4:05), develops febrile neutropenia (38:40), and experiences such severe pain that she is unable to speak in full sentences (1:11:39). In *Terms of Endearment*, Emma Greenway-Horton is diagnosed with breast cancer and admitted to hospital for treatment. Viewers gain a sense of her pain management needs via the advocating that her mother Aurora does on her behalf to the nursing staff. In *Shadowlands*, based upon C.S. Lewis’ book A Grief Observed first published under his pseudonym, Joy Gresham is diagnosed with metastatic cancer and suffers from pain related to a pathological femur fracture. She is shown receiving treatment in hospital as well as convalescing at home. Joy feels guilty about the extra care that her diagnosis will entail for her partner C.S. “Jack” Lewis. In *The Barbarian Invasions*, college professor Rémy has liver cancer and is shown coping with his pain after admission to a Québec hospital. 

#### 3.1.2. Cancer Pain as Affecting the Lives of Those Close to Characters with Cancer

Some films also provide the perspective of the effect of characters’ pain on the lives of those around them. *Shadowlands* shows how Joy’s cancer and its associated pain affects Jack and their son Douglas. So profound is the experience that it leads Jack to reevaluate his spiritual beliefs. In one scene early in the film, before he has met and married Joy, Jack delivers a lecture at Oxford University and characterizes the purpose of pain in this way:

Does God want us to suffer? What if the answer to that question is yes? See I’m not particularly sure that God wants us to be happy…I suggest to you that it is because God loves us that he makes us the gift of suffering. Or to put it another way, pain is God’s megaphone to rouse a deaf world. You see, we are like blocks of stone out of which the sculptor carves the form of men. The blows of his chisel which hurt us so much are what makes us perfect (11:11).

After Joy is admitted to a hospital in London, though, Jack questions God’s intentions:

Yesterday, a friend of mine, a very brave, good woman, collapsed in terrible pain. One minute she was fit and well, next minute she was in agony. She’s now in hospital and this morning I was told she’s suffering from cancer. Why? See, if love someone you don’t want them to suffer. You can’t bear it. You want to take their suffering onto yourself. If even I feel like that, why doesn’t God? (1:10:17).

“Is this pain really necessary?” he asks a nurse in the hospital as Joy writhes in discomfort in her hospital bed (1:16:37). After Joy’s death, Jack questions aspects of his faith. Leaving a church after Joy’s funeral ceremony, colleague Harry Harrington tries to console Jack with his sense of faith: “Thank God for your faith, Jack. Is only faith makes any sense in times like this, I know” (1:58:01). But sitting with his brother Warnier once back at home, Jack is not so sure:

- Jack: What’s happening to me, Warnie?...I’m so afraid…of thinking that suffering is just suffering after all, no cause, no purpose, no pattern.
- Warnie: I don’t know what to tell you, Jack.
- Jack: Hmm? Nothing. There’s nothing to say. I know that now. I’ve just come up against a bit of experience, Warnie. Experience is a brutal teacher, but you’ll learn. My God, you’ll learn (1:58:33).

Meeting with his Oxford colleagues who offer their condolences, Jack’s questioning of his faith is made apparent:

- Harry: Only God knows why these things have to happen.
- Jack: God knows but does God care?
- Harry: Of course. We see so little here. We are not the Creator.
- Jack: No, no. We’re the creatures aren’t we, really? We’re the rats in the cosmic laboratory. I have no doubt the experiment is for our own good, but it still makes God the vivisectionist, doesn’t it?
- Harry: Jack…
- Jack: NO! This is blood awfulness and that’s all there is to it (2:00:16).

*The Barbarian Invasions* provides a different perspective of the effects of a character’s pain on family members by showing the extents to which family goes to try to help their loved one’s pain and suffering. After learning of his estranged father’s cancer diagnosis and associated pain, Sébastien employs a variety of strategies to attend to Rémy’s care. For example, he arranges for Rémy to have a positron emission tomography study expeditiously performed in Vermont because the wait for this is so long in Québec. He bribes a hospital administrator (25:11) and the union facilities management (26:49) to facilitate the creation of a private room for his father’s care in the hospital. He liaises with detectives on a narcotics squad to try to procure heroin to better help with his father’s pain when it seems like the hospital is not helping (36:32). He convinces a nurse to help with his father’s end-of-life care at a lakeside cottage (1:38:42). 

### 3.2. Depictions of Healthcare Professionals Looking after Patients with Pain

In some of the films reviewed, physicians do not appear to be attuned to the needs of the characters with pain. This is seen when physicians are not well-prepared for therapeutic encounters. For example, in *The Barbarian Invasions*, a group of physicians come to evaluate Rémy’s pain. They appear unprofessional: they do not recognize that Rémy is not actually their patient, one of the physicians is eating an apple during the clinical encounter, and they are oblivious that Rémy’s pain management is much better because he had been smoking heroin in the hospital:

- Lead physician: Morning.
- Rémy: Good morning!
- Lead physician: It really smells like cigarette smoke in here. You haven’t been smoking I hope?
- Rémy: Oh no never! I light a candle to meditate.
- Lead physician: You aren’t having any IV solutions?
- Rémy: Dr. Lévesque had it removed. (Turns toward and addresses the other two physicians in the room) Good morning, my doctor-esses.
- Lead physician: Take a deep breath. Does it hurt here?
- Rémy: Oh, yes.
- Lead physician: And here, too?
- Rémy: There too, yes.
- Lead physician: Is it unbearable?
- Rémy: Goodness, no!
- Lead physician: You’re in high spirits.
- Rémy: Couldn’t be higher!
- Lead physician: How do you sleep?
- Rémy: Like a baby.
- Lead physician: Then I won’t prescribe painkillers.
- Rémy: Forget it!
- Lead physician: That’s wonderful. I always say, the longer they stay lucid, the better.
- Rémy: I plan to remain lucid til I die.
- Lead physician: Wonderful, Mr. Parenteau.
- Rémy: Thanks so much, Dr. Dubé.
- Lead physician: I’m not Dr. Dubé.
- Rémy: How fitting, because I’m not Mr. Parenteau! (49:49).

Physicians sometimes appear callous in their interactions with patients with pain. In *Wit*, Professor Bearing’s oncologist, Dr. Kelekian, delivers her diagnosis abruptly—the abruptness is accentuated by the piercing music and sudden appearance of Dr. Kelekian on the screen in this opening scene. She undergoes a degrading pelvic examination from Dr. Jason Posner, Dr. Kelekian’s fellow. On physician rounds, a group of physicians suddenly enter Professor Bearing’s room. The blankets are quickly lifted away exposing her lower body. The physicians rudely push on her abdomen. Professor Bearing is barely spoken to by the physicians and is spoken about as if she is merely a constellation of medical abnormalities. “They anatomize me”, she tells the audience (38:23). Later in the film when Professor Bearing is writhing in bed in such severe pain that she has difficulty speaking, Dr. Kelekian seems dismissive and paternalistic in his approach to her care. Professor Bearing looks at the camera and speaks directly to the audience in disbelief when she’s asked by Dr. Kelekian if she’s having pain, as if it was not obvious. His behaviour stands in contrast to that of Susie, Professor Bearing’s nurse, who appears compassionate and acts as an advocate for her pain management:

- Dr. Kelekian: Dr. Bearing.
- Susie: It’s time for patient-controlled analgesia. The pain is killing her.
- Dr. Kelekian: Dr. Bearing are you in pain?
- Prof. Bearing: I don’t believe this.
- Dr. Kelekian: I want a morphine drip.
- Susie: What about patient-controlled? She could be more alert?
- Dr. Kelekian: Ordinarily, yes. But in her case, no.
- Susie: B-but I think she would really rather…(she looks down and away from Dr. Kelekian, as if embarrassed).
- Dr. Kelekian: She’s earned a rest. Morphine. Ten push now and start at 10 an hour. Dr. Bearing, try to relax. (Susie looks at Dr. Kelekian with a look of incredulousness) (1:13:36).

The empathy shown by Susie is accentuated by the film’s depiction of the lack of empathy shown by the physicians. When Dr. Posner sees Dr. Bearing lying in her bed in extreme pain, he looks at her from the foot of the bed, seemingly not knowing what to say or do. He puts his hands in his jacket pockets, slumps his head forward, and walks away (1:14;22). He does not utter even one word of empathy or compassion. 

The provision of pain care by the nurse in *Wit* was portrayed in a positive way. For example, nurses were responsive to and advocated for their patient’s pain management. In *Wit*, Susie appears to respond promptly to Professor Bearing’s cries of severe pain; she rushes from the nurses’ station to the bedside and immediately suggests a strategy to help: “just try and relax and clear your mind (she rubs Professor Bearing’s arm). We’ll get you patient-controlled analgesic. It’s a little pump with a little button, and you press it, and you decide how much medication you want. It’s very simple, and it’s all up to you (1:13:14). As Professor Bearing receives morphine from Susie, we see them sharing a laugh that conveys a sense of humanity that the nurse brings to the situation:

- Prof. Bearing: I trust this will have a soporific effect.
- Susie: I’m not sure about that but it sure does make you feel sleepy (1:15:43).

The scene closes with the camera panning out to show Professor Bearing and Susie in good spirits as Susie draws the curtain to the room. 

In *The Barbarian Invasions*, a nurse recognizes Rémy’s suffering and confronts ethical Issues related to his care by acting to relieve his suffering. After Rémy leaves the hospital to go to the Québec countryside, Nurse Carole drives to the cottage to facilitate his euthan asia, despite that being beyond the scope of her practice and nursing jurisprudence. She humours Rémy’s comments to her in a caring way as they share a smile (1:38:15). 

At times, however, nurses were depicted as being too busy to attend to patients’ needs. In *Terms of Endearment*, Aurora Greenway becomes frustrated when the nurses are not attentive to pain management for her daughter:

- Aurora: Excuse me. It’s after ten, give my daughter the pain shot please.
- Nurse 1: Mrs. Greenway. I was going to.
- Aurora: Oh! Good. Go ahead.
- Nurse 1: Just a few minutes (with her voice raised and open hand up).
- Aurora: Well please! It’s after 10, it’s after 10. I don’t see why she has to have this pain (with raised voice).
- Nurse 2: Ma’am, it’s not my patient.
- Aurora: It’s time for her shot! YOU UNDERSTAND? DO SOMETHING! ALL SHE HAS TO DO IS HOLD OUT UNTIL 10 AND IT’S PASSED 10, SHE IS (gasps) IN PAIN, MY DAUGHTER’S (gasps) IN PAIN, GET HER THE SHOT! DO YOU UNDERSTAND?
- Nurse 1: You are going to have to behave, Mr. Greenway.
- Aurora: GIVE MY DAUGHTER THE SHOT!

In *The Barbarian Invasions*, the nurses at times are too overworked to attend to patients. 

“I’ve been on duty for 12 h without a break. I’m doing three people’s work”, a nurse tells Sébastien (9:41), “so go bother someone else” (9:48). 

### 3.3. Depictions of Therapies for Pain Management

Opioids are the most common medication used for pain management in the reviewed films. No other types of pain management therapies are depicted in any of the films. In *Wit*, Professor Bearing is administered a morphine infusion and the following scene shows her much more comfortable. The scene ending with Susie closing the door to Professor Bearing’s room and drawing the curtains is symbolic as this is one of the last scenes in which the audience sees Professor Bearing awake and able to communicate. The initiation of the morphine infusion heralds the drawing of the curtain on her life. She thenceforward appears sedated or unresponsive in bed: “like she can hear you”, Dr. Posner says to Susie as they place a Foley catheter in her (1:18:26). 

Rémy resorts to smoking heroin to manage his pain. Heroin use is depicted as being secondary to his inability to obtain proper pain management in the hospital. Sébastien resorts to approaching the police for information on how to obtain heroin for his father’s pain but ultimately is able to source some from Nathalie, the daughter of one of Rémy’s former lovers who has an opioid misuse problem. Once Rémy is able to use heroin, he is seen as being much more comfortable. 

### 3.4. Depictions of the Settings for Pain Management

The settings of some of the films also speak to the challenges that characters experience obtaining pain management. This is especially clear in *The Barbarian Invasions*. As if to give a sense of the state of the healthcare system in Québec, and a premonition of the challenges of obtaining care, the film opens by following a nurse as she walks through the hospital hallways. The audience sees crowded corridors: a family member feeds their loved one on a stretcher, patients cross the hallway holding onto hospital poles, and electricians fix wires through holes in the ceiling, for example. After Sébastien’s laptop is stolen in the hospital, a security guard tells him that items are stolen all the time and a woman was raped in the laundry room. 

After the audience is introduced to Rémy, the challenges that this healthcare environment pose to his ability to obtain care become obvious as well. He is initially in a crowded room. At night, one of the other patients watches television so loudly that it is audible to everyone in the room. “I’m lucky I’m not in the hall”, Rémy concedes (8:21). He goes to the United States to obtain more prompt imaging studies. A hospital administrator accepts a bribe to facilitate the setting up of a private room for him. As if to acknowledge that the pain management available in the hospital is insufficient, a physician suggests that Sébastien obtain heroin for his father’s pain. Thus, the state of an overextended healthcare system is laid bare to be seen. The film thus asks: how can patients obtain proper pain care in such a setting?

## 4. Discussion

This is the first study to review films featuring characters with cancer pain and to explore how those characters and their pain management are portrayed. The films reviewed show characters’ pain experiences, their experiences with the healthcare system and healthcare professionals, and how their pain was managed. Since the films reviewed are easily accessible to the public and have received accolades such as Academy Awards and Golden Globes, there is the potential that these portrayals can influence the public perception of cancer pain and its management. That possibility lends itself to the use of such film in the education of healthcare professionals regarding cancer pain. 

### 4.1. Depictions of Characters with Cancer Pain

In some ways, the portrayal of characters’ experiences with pain bears semblance to the lived experiences of patients. The films show that cancer can cause significant pain and affect patients’ quality of life. It is not clear whether realistic portrayals of illness in film can decrease stigma that may be associated with them. For example, studies have suggested that stigma-related attitudes about schizophrenia did not change [63] or even worsened [64] after viewers watched films depicting the condition realistically. Röhm and colleagues suggest that this may be the case because realistic portrayals may disturb viewers such that they distance themselves even further from them [65]. 

### 4.2. Depictions of Healthcare Professionals’ Attitudes

The physicians in some of the reviewed films do not seem to be attentive to the pain management needs of the characters. Physicians misrecognize Rémy, do not recognize the type of pain management he is receiving, and appear unprofessional in their interaction with him. Dr. Kalekian and Dr. Posner seem more concerned about Professor Bearing from their research perspective than as their patient and their degree of empathy stands in stark contrast to compassion of her nurse Susie. Physicians are not immune to biases that contribute to patients’ concerns about their pain management [66,67]. Poor physician communication has been shown to lead to incongruity between patients’ and their physicians’ expectations for pain management [68,69]. In some ways, then, the resignation that some of the characters in the films reviewed expressed towards interacting with physicians is not surprising.

In contrast, some of the depictions of nurses in the reviewed films were complementary of their role in pain management. Nurses are recognized to have instrumental roles in providing pain care [70,71]. Nurses identify poor communication with physicians as among the barriers to their ability to provide effective pain care [72,73]. This was well-exemplified in *Wit*. Van Niekerk and colleagues [73] found that there was a significant interaction between nurses’ feeling that they were “adequately consulted by physicians” and “the perceived barrier to pain management” (p. 8). When nurses felt empowered to help make pain management decisions, their well-being improved as was their sense of feeling prepared to “deliver care that was synonymous with their professional values” [74] (p. 20). They were more apt to liaise with physicians about their patients’ pain needs and potential problems with their pain management [72]. When nurses felt disempowered or dissatisfied with the manner in which physicians communicated or worked with them, they were “more likely to encounter ethical conflicts such as inadequate pain relief (undermedication), patient reluctance to report pain, and conflicting opinions with physicians” [72] (p. 285). 

### 4.3. Depictions of Pain Management Strategies

Opioid use came across in some of the reviewed films as being the only available pain management therapy. In other cases, it was a treatment of last resort. Such is the case in *The Barbarian Invasions* in which the protagonist uses heroin because of few other available options. In *Wit*, opioid use is associated with succumbing to cancer and suffering. Once Professor Bearing’s pain is treated with opioids, the film soon depicts her as falling into a coma. 

### 4.4. Depictions of Pain Management Settings

Studies have demonstrated that the setting in which patients obtain pain management influences the quality of their care [70,75,76,77]. For example, it has been shown that the organizational structure within healthcare systems can influence how pain management knowledge is disseminated and how pain management strategies are implemented [70]. The films reviewed depict this in different ways. First, it is recognized that institutional culture can affect the relationship between their healthcare providers and staff, and this in turn can affect the manner in which they interact with patients [75,78]. This was particularly clear in *Wit* in the interactions between the physicians and nurse looking after Professor Bearing. Physicians’ interactions were typically short, brusque, and lacking in empathy. Physicians were seen as leaving any humanistic elements of patient care to the nurse, such as offering a tissue to Professor Bearing after a pelvic examination. It is therefore not surprising to see that cultural hierarchy becoming manifest in those providers’ discussion of and approach to pain management. Second, Fagerhaugh and Strauss [78] point out that the acute care model is dominant in hospitals but may not be well-suited to the management of issues like cancer pain. Indeed, none of the characters in the films reviewed are seen as receiving multidisciplinary pain care. Last, the depiction of an overburdened healthcare system may lead audiences to see the barrier that this poses for patients to receive adequate and timely pain care. Healthcare providers are represented as being confined to institutional rules and structures and helpless and overwhelmed by systemic inadequacies. Films like *The Barbarian Invasions* and *Terms of Endearment* demonstrate how the nurses seem to have no time to attend to patients’ needs. Many of the nurses in the study by Morgan and colleagues [75] echoed the sentiment that they were nursing the computer instead of having enough time in a shift to attend to patients’ pain. Other concerns noted included staffing shortages and documentation hurdles [75]. In a hospital that is literally falling apart, care for Rémy can only be obtained by going to another country to obtain imaging studies, bribing hospital administrators and construction workers, approaching narcotic police officers for access to heroin, arranging for someone to provide heroin, and ultimately going to the countryside to obtain comfort. 

### 4.5. Implications for Improving Cancer Pain Education with Cinemeducation

To date, the use of cinemeducation as a tool in cancer pain management education has not been described. Review of these Hollywood films suggests that there is content that could be used to help educate healthcare professionals about different aspects of cancer pain management. For example, the films can be used to prompt students to think about the way in which pain affects patients’ experiences with cancer. Studies related to oncology and palliative care education suggest that the promotion of caring attitudes towards patients with pain as well as improving communication skills are important pedagogical objectives [79,80,81]. To that end, the reviewed films lend themselves well to teaching students about these topics by asking the students to reflect on scenes in which healthcare professionals interact with characters with pain. 

The proof-of-principle that the humanities can be effectively incorporated into cancer pain curricula has been demonstrated [28,82]. At the University of Parma School of Oncology, works of literature have been incorporated into teaching programs to enhance physicians’ and nurses’ abilities to communicate with patients. At the University of California at Los Angeles, the play “Wit” has been used to provide physicians and physicians-in-training with end-of-life education [83]. The program was expanded to medical centres throughout North America [83]. Kumagai [34] has argued that humanities-based educational programs, such as these, provide a “transformative” (p. 656) educational experience because they allow “glimpses into the subjective world of lived experience, forging emotional links with the other, stimulating self-reflection through cognitive dissonance, and eliciting resonance of similar, fundamental emotions in the learner” (p. 657).

### 4.6. Limitations

It is possible that not all films depicting cancer pain were found. For this study, Hollywood films were selected for review and so this study is biased towards themes that emerge from a predominantly contemporary North American perspective. Review of films from other countries might provide different perspectives and themes related to cancer pain management. Moreover, the films reviewed include characters who might primarily identify in society as white-cis-het; films that contain characters who self-identify in other ways might provide different perspectives as well. It is also acknowledged that the interpretation of films is subjective and so the themes presented in this study are not exhaustive. 

## 5. Conclusions

This study demonstrates that patients with cancer pain featured in Hollywood films are depicted in a compassionate manner. Pain management in these films focused on the use of opioids. The settings in which patients received pain management was depicted as not being amenable to providing holistic care. This variety of topics related to pain management covered in the films make them amenable to use in cinemeducation. This study therefore provides a foundation for future work looking at the depiction of cancer pain and end-of-life care in films and their potential use in health professional education. This would be important to help elucidate how cinemeducation could be incorporated into existing pain management curricula, which disciplines could be involved cinemeducation-based teaching modules, and which stages of healthcare professionals’ training would be best-suited to having this educational exposure. 

## Figures and Tables

**Table 1:** Films analyzed for depictions of cancer pain and its management.

Film	Year of Release	Box Office Earnings	Selected Awards Won
Terms of Endearment	1983	$108,423,749	***Academy Awards:***
			Best Picture, Best Director, Best Adapted
			Screenplay, Best Actress, Best Supporting Actor
			*Golden Globe Awards:*
			Best Motion Picture—Drama,
			Best Actress in a Motion Picture—Drama,
			Best Supporting Actor—Motion Picture,
			Best Screenplay—Motion Picture
Shadowlands	1993	$25,842,377	***Academy Awards:***
			Nomination for Best Actress,
			Nomination for Best Adapted Screenplay
Wit	2001	not applicable	***Golden Globes:***
			Best Mini-series or Motion Picture Made for
			Television, Best Performance by an Actress in a
			Mini-Series or Motion Picture Made for
			Television
			***Primetime Emmy Award:***
			Outstanding Made for Television Movie,
			Outstanding Directing for a Miniseries, Movie
			or a Special, Outstanding Lead Actress,
			Outstanding Support Actress, Outstanding
			Writing for a Miniseries, Movie or Dramatic
			Special
The Barbarian Invasions	2003	$34,883,010	***Academy Awards:***
			Best Foreign Language Film,
			Nomination for Best Original Screenplay
			***Golden Globes:***
			Nomination for Best Foreign Language Film
